# A Nationwide Multicenter Study on 1-Year Outcomes of Posterior Chamber Phakic Intraocular Lens Implantation for Low Myopia

**DOI:** 10.3389/fmed.2022.762153

**Published:** 2022-05-04

**Authors:** Kazutaka Kamiya, Kimiya Shimizu, Akihito Igarashi, Yoshihiro Kitazawa, Takashi Kojima, Tomoaki Nakamura, Kazuo Ichikawa, Sachiko Fukuoka, Kahoko Fujimoto, The Japan ICL Study Group

**Affiliations:** ^1^Visual Physiology, School of Allied Health Sciences, Kitasato University, Tokyo, Japan; ^2^Department of Ophthalmology, Sanno Hospital, Tokyo, Japan; ^3^Department of Ophthalmology, Sapia Tower Eye Clinic Tokyo, Tokyo, Japan; ^4^Department of Ophthalmology, Keio University, Tokyo, Japan; ^5^Department of Ophthalmology, Nagoya Eye Clinic, Aichi, Japan; ^6^Department of Ophthalmology, Chukyo Eye Clinic, Aichi, Japan; ^7^Department of Ophthalmology, Tane Memorial Eye Hospital, Osaka, Japan; ^8^Department of Ophthalmology, Fujimoto Eye Clinic, Osaka, Japan

**Keywords:** phakic IOL, safety, efficacy, predictability, stability, endothelial cell density, low myopia, EVO-ICL

## Abstract

**Purpose:**

To assess the nationwide multicenter outcomes of posterior chamber phakic intraocular lens implantation with a central hole (EVO-ICL, STAAR Surgical) for patients with low myopia.

**Methods:**

This multicenter study comprised 172 eyes of 111 consecutive patients undergoing hole ICL implantation to correct low myopia and myopic astigmatism [manifest spherical equivalent (MSE);−3 diopters (D) or less] at seven nationwide major surgical facilities. We retrospectively determined safety, efficacy, predictability, stability, and adverse events at 1 week, 1, 3, 6, and 12 months postoperatively, and at the final visit.

**Results:**

The mean follow-up period was 1.4 ± 1.0 years. Uncorrected and corrected visual acuities at 1 year postoperatively were −0.17 ± 0.12 and −0.24 ± 0.07 logarithm of the minimal angle of resolution (logMAR), respectively. At 1 year postoperatively, 91% and 100% of eyes were within 0.5 and 1.0 D of the target correction, respectively. No significant manifest refraction changes of −0.07 ± 0.26 D occurred from 1 week to 1 year. No vision-threatening complications occurred at any time in this series.

**Conclusions:**

According to our experience, the EVO-ICL performed well without significant complications throughout the 1-year observation period, even for the correction of low myopia. It is suggested that current ICL implantation is one of the viable surgical options for correcting low myopia.

## Introduction

The EVO Visian Implantable Collamer Lens (EVO Visian ICL^TM^, STAAR Surgical, Monrovia, CA, USA), a posterior chamber phakic intraocular lens, is well recognized worldwide as a long-term safe and effective means of correcting moderate-to-severe refractive errors (1–6). However, conventional ICL implantation has several disadvantages over keratorefractive surgeries in the necessity of preoperative laser iridotomy or intraoperative peripheral iridectomy to prevent the occurrence of pupillary block and the possible risk of cataract formation. The ICL with a central port (KS-AquaPORT V4c and V5; EVO-ICL) was developed to rectify such drawbacks without significant deterioration of visual performance (7–9). Moreover, effective correction of moderate-to-severe myopia has been reported (10–14). In addition, ICL surgery is primarily reversible and allows ICL exchange, unlike laser *in situ* keratomileusis (LASIK), even when unexpected outcomes occur postoperatively (15).

Hitherto, the surgical indication for ICL implantation has still been generally thought to be indicated for eyes with a moderate-to-severe myopia. However, several studies have shown promising results of ICL implantation for low-to-moderate myopia (16–19). Indeed, according to the Japanese guidelines for phakic intraocular lens implantation, the surgical indication of myopic ICL implantation has been previously indicated to be used with caution for myopia of −6 to −15 diopters (D) and myopia of −15 D or greater, but has recently expanded to be used with caution for myopia of −3 to −6 D. Furthermore, based on our findings that ICL implantation induces significantly fewer higher-order aberrations than wavefront-guided LASIK and that the contrast sensitivity function was significantly improved after ICL implantation but unchanged after wavefront-guided LASIK even in low-to-moderate myopic eyes (20), the surgical indication for ICL implantation might be further expanded to include low myopic eyes. However, there are no studies on the detailed outcomes of current ICL implantation for low myopic eyes (3 D or less) only, possibly because of the limited number of ICL surgeries for such myopia. Therefore, it may provide intrinsic insights into further expanding this surgical indication of ICL surgery in the future. The present study aims to retrospectively assess the clinical outcomes of current ICL implantation for low myopia in a large cohort of patients attending major surgical facilities in Japan. This multicenter study was held under the auspices of the Japan ICL Study Group. To our knowledge, this is the first nationwide multicenter case series to investigate the outcomes of current ICL implantation only in eyes having low myopia.

## Materials and Methods

### Study Population

The protocol is registered in the University Hospital Medical Information Network Clinical Trial Registry (000045089). Consecutive patients who underwent implantation of the posterior chamber phakic ICL with a 0.36-mm central port (EVO-ICL) for the correction of low myopia and myopic astigmatism (manifest spherical equivalent (MSE); 3 D or less) at seven major nationwide institutions (Kitasato University Hospital, Sanno Hospital, Sapia Tower Eye Clinic Tokyo, Nagoya Eye Clinic, Chukyo Eye Clinic, Tane Memorial Eye Hospital, and Fujimoto Eye Clinic) from January 2016 to June 2020 and who completed a 1-year follow up were retrospectively assessed by the review of the clinical charts at each institution. The primary inclusion criteria for ICL surgery include unsatisfactory correction with spectacles or contact lenses, 20 ≤ age ≤ 50 years at the time of surgery, stable refraction, astigmatism of 3 D or less, anterior chamber depth (ACD) ≥ 2.8 mm, and endothelial cell density (ECD) ≥ 1,800 cells/mm^2^. Any history of ocular surgery, corneal diseases, including keratoconus and pellucid marginal degeneration, cataract, glaucoma, uveitis, other concomitant eye diseases, or intentional undercorrection or monovision in middle-aged patients, were excluded from the study. The targeted refraction was set at emmetropia in all eyes. The study was approved by the Institutional Review Board at Kitasato University Hospital (identifier: B21-195) and followed the tenets of the Declaration of Helsinki. Written informed consent for ICL surgery was obtained from all patients after explaining the possible consequences.

### Outcomes Measures

Preoperatively, 1 week, 1, 3, 6, and 12 months postoperatively, and at the last visit (spanning more than 1 year), we determined the following metrics: the logarithm of the minimal angle of resolution (logMAR) of uncorrected distance visual acuity (UDVA) and corrected distance visual acuity (CDVA), the MSE, the intraocular pressure (IOP) using a non-contact tonometer, the ECD (preoperatively and 1 year postoperatively) using a non-contact specular microscope, and the vault between the anterior crystalline lens surface and the posterior ICL surface using an anterior segment optical coherence tomography (AS-OCT), in addition to routinely performed ophthalmic examinations. All available visit data were collected and grouped according to the closest time point. If more than one visit was available within a given grouping, we used the visit data most comparable to the given time point for this analysis.

### Power Calculation and Size Selection

We determined the ICL size (12.1, 12.6, 13.2, and 13.7 mm), mainly based on the manufacturer's nomogram using the white-to-white (WTW) distance and the ACD using a scanning-slit light corneal tomographer (Orbscan, IIz, Bausch&Lomb, Rochester, USA) or an AS-OCT (CASIA, Tomey Corporation Co Ltd, Aichi, Japan). We also selected the ICL power using an online calculation and ordering system provided by the manufacturer based on a modified vertex formula (21, 22). We basically selected the toric model ICL in eyes with manifest astigmatism of 1 D or more and the nontoric model ICL in eyes with astigmatism below 1 D.

### Surgical Procedures

We described the details of the surgical procedures in our preceding reports (10–12, 18). In brief, on the day of surgery, dilating and topical anesthetic agents were applied. The model V4c or V5 ICL was implanted through a 3- to 3.2-mm temporal clear corneal incision after the injection of a viscosurgical substance into the anterior chamber. Next, the ICL was inserted into the posterior chamber, the viscosurgical substance was replaced with a balanced salt solution, and a miotic agent was administered. We topically utilized antibiotic and steroidal medications four times a day for 1 week, and the dose was gradually reduced.

### Statistical Analysis

The normality of all data samples was first checked using the Shapiro–Wilk test. As all data fulfilled the criteria for normal distribution, the paired *t*-test was used to compare the pre-surgical and postsurgical data. Fisher's exact test was used to compare the percentages between the two groups. One-way analysis of variance (ANOVA) was used to assess the time-course of changes, with the Dunnett test employed for multiple comparisons. The Pearson correlation coefficient was used to assess the relationship between the two variables. Unless otherwise indicated, the results are expressed as mean ± standard deviation (SD) [95% confidence interval (CI)], and a value of *p* < 0.05 was considered statistically significant.

## Results

### Study Population

A total of 172 eyes of 111 patients (93 of men and 79 of women) met the inclusion criteria of this study. Nine (8%) patients were lost during a follow-up, due to a transfer to another clinic, work responsibilities, and distance. The mean follow-up period was 1.4 ± 1.0 years. Table 1 shows the preoperative baseline demographics of the study population.

**Table 1:** Preoperative demographics in eyes undergoing implantable Collamer lens (ICL) implantation for low myopia.

**Characteristic**	**Mean ±standard deviation (95%CI)**
Age	33.8 ± 6.8 years (95%CI, 20 to 46.5 years)
Gender	Male: Female = 93: 79
Manifest spherical equivalent	−2.28 ± 0.56 D (95%CI, −1.20 to −3.37 D)
Manifest cylinder	−0.98 ± 1.36 D (95%CI, −3.65 to 1.69 D)
LogMAR UDVA	0.73 ± 0.29 (95%CI, 0.16 to 1.30)
LogMAR CDVA	−0.23± 0.08 (95%CI, −0.39 to −0.06)
White-to-white distance	11.9 ± 0.4 mm (95%CI, 11.1 to 12.8 mm)
Anterior chamber depth	3.21 ± 0.30 mm (95%CI, 2.61 to 3.80 mm)
Mean keratometric readings	43.40 ± 1.44 D (95%CI, 40.57 to 46.23 D)

*ICL, implantable collamer lens; CI, confidence of interval; D, diopter; LogMAR, logarithm of the minimal angle of resolution; UDVA, uncorrected distance visual acuity; CDVA, corrected distance visual acuity*.

### Safety and Efficacy Outcomes

**Figure 1:**
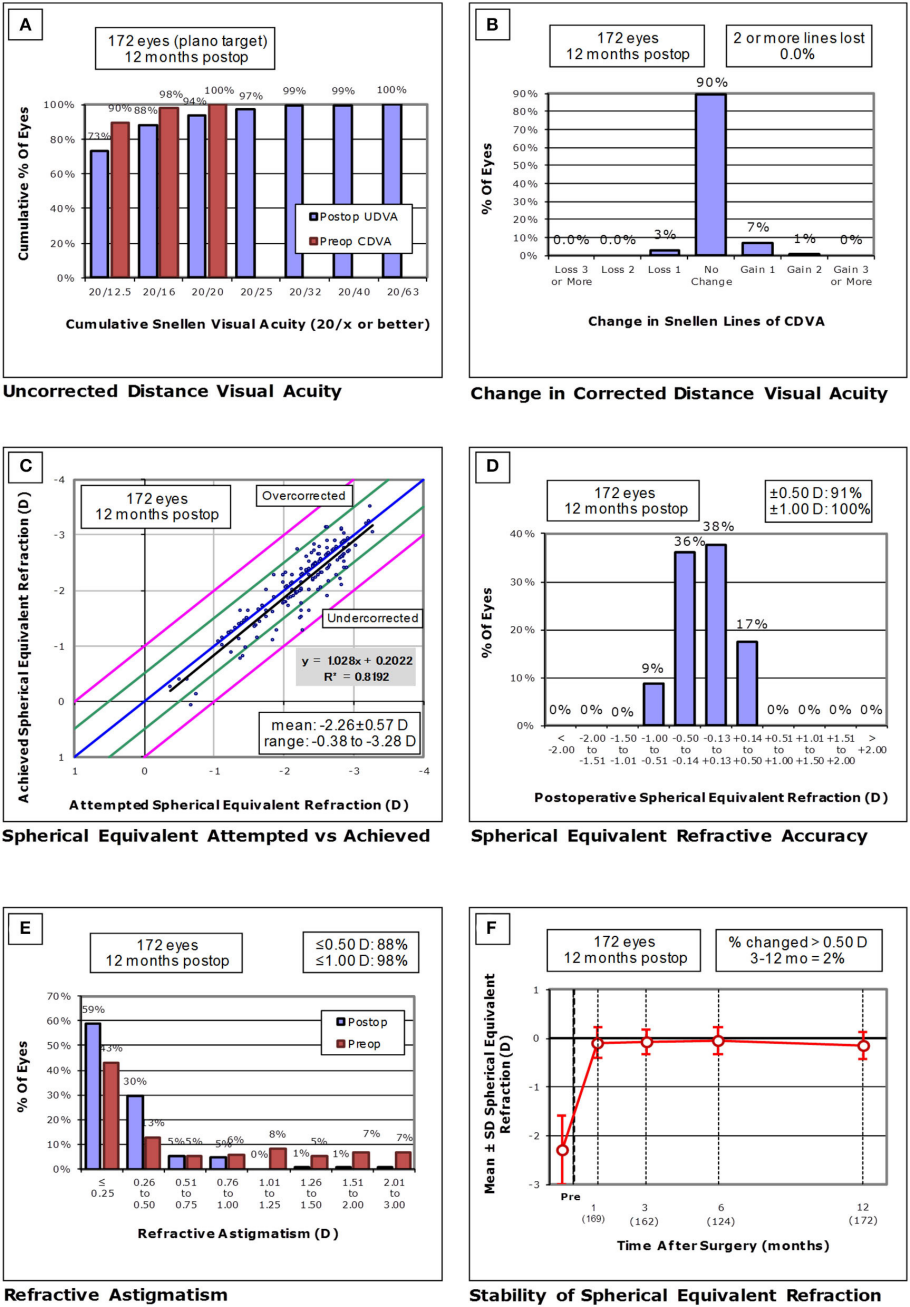
**(A)** Cumulative percentages of eyes attaining specified cumulative levels of uncorrected distance visual acuity (UDVA), **(B)** Changes in corrected distance visual acuity (CDVA), **(C)** A scatter plot of the attempted vs. the achieved manifest spherical equivalent (MSE) correction, **(D)** Distribution of spherical equivalent refractive accuracy, **(E)** Distribution of refractive astigmatism, and **(F)** Time course of changes in MSE.

At 1 week, 1, 3, 6, and 12 months postoperatively, and the last visit, 95, 96, 96, 94, 94, and 77% of eyes, and 72, 70, 73, 72, 73, and 57% of eyes, respectively, had a UDVA of 20/20, and 20/16 or better (Figure 1A). LogMAR UDVA was −0.16 ± 0.12, −0.17 ± 0.11, −0.18 ± 0.11, −0.18 ± 0.11, −0.17± 0.12, and −0.10 ± 0.17, at 1 week, 1, 3, 6, and 12 months postoperatively, and the last visit, respectively. We found a significant difference between preoperative UDVA and 1-year postoperative UDVA (*p* < 0.001). The efficacy index (mean postoperative UDVA/mean preoperative CDVA) was 0.91 ± 0.20 at 1 year postoperatively.

At 1 year postoperatively, 154 eyes (90%) showed no change in CDVA, 12 eyes (7%) gained 1 line, and 1 eye (1%) gained 2 lines, and 5 eyes (3%) lost 1 line, but no eyes had lost more than 1 line (Figure 1B). LogMAR CDVA was −0.23 ± 0.08, −0.23 ± 0.08, −0.24 ± 0.07, −0.24 ± 0.08, −0.24 ± 0.07, and −0.22 ± 0.08, at 1 week, 1, 3, 6, and 12 months postoperatively, and the last visit, respectively. We found a significant difference between preoperative CDVA and 1-year postoperative CDVA (*p* = 0.003). The safety index (mean postoperative CDVA/mean preoperative CDVA) was 1.06 ± 0.20 at 1 year postoperatively.

### Predictability and Stability Outcomes

A scatter plot of the attempted vs. the archived MSE correction, the distribution of spherical equivalent refractive accuracy, and the distribution of refractive astigmatism are shown in Figures 1C–E, respectively. At 1 week, 1, 3, 6, and 12 months postoperatively, and the last visit, 95, 96, 97, 94, 91, and 77% of eyes, and 100, 99, 100, 100, 100, and 93% of eyes were within ± 0.5 and 1.0 D, respectively, of the attempted spherical equivalent correction.

The time-course change in the MSE is shown in Figure 1F. At 1 week, 1, 3, 6, and 12 months postoperatively, and the last visit, the MSE was −0.07± 0.28, −0.09 ± 0.31, −0.07 ± 0.25, −0.05 ± 0.28, −0.14 ± 0.28, and −0.22 ± 0.51 D, respectively (one-way ANOVA, *p* = 0.396). The changes in MSE refraction from 1 week to 1 year were −0.07 ± 0.26 D.

### Intraocular Pressure

**Figure 2:**
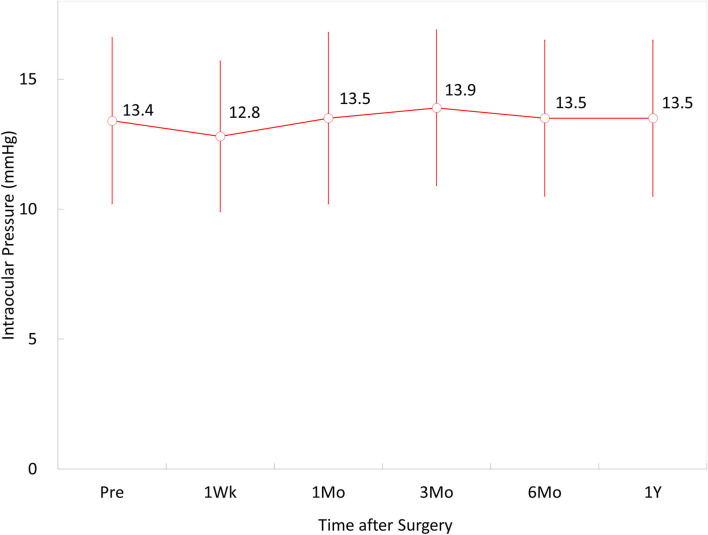
Time course of changes in intraocular pressure (IOP) after implantable Collamer lens (ICL) implantation in eyes with low myopia.

The IOP was 13.4 ± 3.2, 12.8 ± 2.9, 13.5 ± 3.3, 13.9 ± 3.0, 13.5 ± 3.0, and 13.5 ± 3.6 mmHg, at 1 week, and 1, 3, 6, and 12 months postoperatively, and the last visit, respectively (*p* = 0.114) (Figure 2). Throughout the observation period, no significant increase in the IOP (>25 mmHg) occurred in any case.

### Endothelial Cell Density

The ECD changed, but not significantly, from 2,791 ± 248 cells/mm^2^ preoperatively to 2,756 ± 241 cells/mm^2^ at 1 year postoperatively (*p* = 0.203). The mean percentage of endothelial cell loss was 0.8% ± 6.1% at 1 year postoperatively.

### Vault

**Figure 3:**
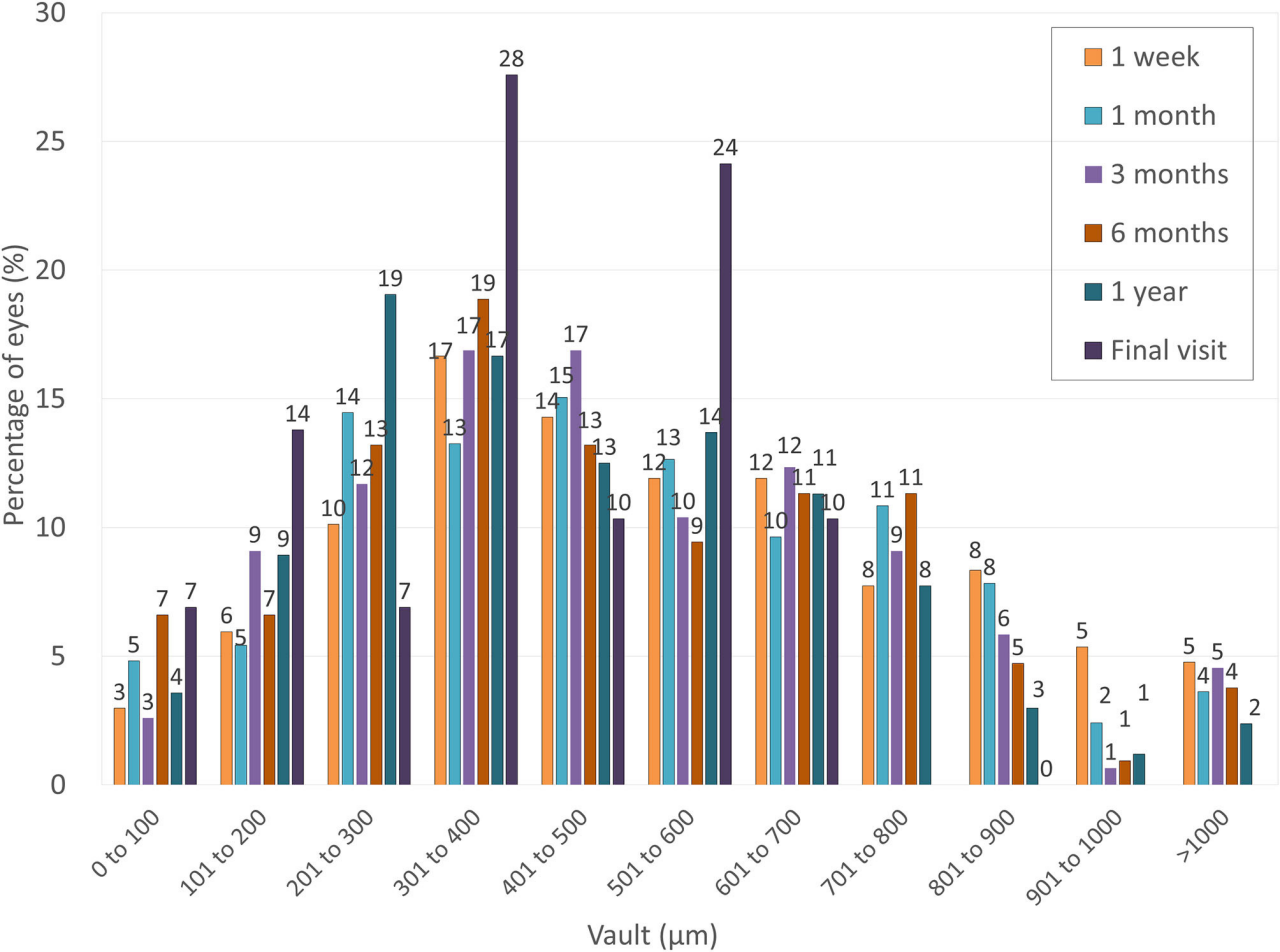
Distribution of eyes according to the vault after ICL implantation in eyes with low myopia.

The ICL vault was 538 ± 271, 506 ± 268, 490 ± 257, 477 ± 268, 447 ± 238, and 379 ± 186 μm, at 1 week, and 1, 3, 6, and 12 months postoperatively, and the last visit, respectively (*p* = 0.005). Multiple comparisons demonstrated significant differences between measurements made at 1 week and 1 year (*p* = 0.006) and at 1 week and the last visit postoperatively (*p* = 0.010). Figure 3 shows the postoperative distribution of the ICL vault. We found a significant correlation between the ICL vault and the MSE at 1 year postoperatively (Pearson's correlation coefficient *r* = 0.213, *p* = 0.006). Throughout the observation period, neither excessively low vault (<50 μm) nor excessively high vault (>1,250 μm) requiring ICL exchange was not found in any case.

### Secondary Surgeries/Adverse Events

We found no apparent intraoperative complications, such as an upside-down ICL insertion or traumatic cataract formation, and all ICL implantations were uneventful in this series. One eye (0.6%) required ICL lens exchange with a different power due to undercorrection at 1 week postoperatively, and UDVA was improved from 20/40 to 20/20 after ICL exchange. Four eyes (2.3%) required rerotation of toric ICLs. Four eyes (2.3%) complained of symptomatic glare or halo during the early postoperative period, but these symptoms recovered over time. Otherwise, we found no vision-threatening complications, such as symptomatic or asymptomatic cataract formation, pigment dispersion glaucoma, pupillary block, retinal detachment, or significant endothelial cell loss (≥20%), throughout the observation period in any case.

## Discussion

In this study, our multicenter results confirmed that current ICL implantation is good in terms of safety, efficacy, predictability, and stability, even for the correction of low myopia and myopic astigmatism, and that no significant intraoperative or postoperative complication occurred in any cases throughout the 1-year observation period. Our findings suggest that ICL implantation can be one of the viable surgical options to correct not only moderate-to-severe myopia but also for low myopia. As far as we can ascertain, there have been no detailed studies on the single outcomes of current ICL implantation for limited eyes with low-grade myopia (3 D or less) consistent with the contemporary notion of low myopia. We suggest that this information is clinically beneficial to reconsider the surgical indication and the prevalence of this promising treatment for all degrees of myopic refractive errors. The surgical procedure of ICL implantation for low myopia was not essentially different from that for moderate-to-severe myopia. However, patient selection was somewhat different because both ICL implantation and LASIK can be performed especially for low-to-moderate myopia in most cases. Therefore, we usually discussed the pros and cons of these surgeries, including cost-effectiveness, visual and refractive outcomes, and long-term prognosis, with such candidates, and finally determined the individual surgical plan when both surgeries were indicated.

Until now, there have been several published studies on the outcomes of ICL implantation for low-to-moderate myopia, as summarized in Table 2 (16–19). Sanders et al. firstly, reported that ICL implantation had advantages over LASIK even in eyes with low myopia (16). Sanders et al., later, showed that ICL implantation was superior to LASIK regarding safety, efficacy, predictability, and stability, for myopia of −3.00 to −7.88 D with matching preoperative data (17). However, the range of myopia is −4 to −8 D and −3 to −7.88 D, and the mean MSE is −6.4 ± 1.1 D and −6.01 ± 1.40 D in their studies, all of which may be somewhat different from the current understanding of the range and the degree of low-to-moderate myopia. We previously demonstrated that ICL performed equally well in correcting low-to-moderate myopia (<6 D) as it did in high myopia (6 D or more) during the 1-year observation period. Recently, Pinto et al. showed comparable 1-year postoperative safety, efficacy, predictability, and stability results for patients with low (6 D or less) and moderate-to-severe (more than 6 D) myopia (16). It should be noted that all previous studies included patients with MSE <-8 D (16, 17), <-6 D (18), or −6 D or less (19), as a definition of low or low-to-moderate myopia. Our findings for low myopia were consistent with the previous results for low-to-moderate myopia in almost all outcomes measured. However, the degree of myopia in our study was much less than that in previous studies because we included only eyes with an MSE of −3 D or less.

**Table 2:** Summary for the outcomes of ICL implantation for low-to-moderate myopia.

**Author**	**Year**	**Type**	**Period (months)**	**Eyes**	**Age (years)**	**MSE (D)**	**UDVA**	**CDVA**	**Within ±0.5 D**	**Within ±1.0 D**	**Cataract**
Sanders et al. (16)	2006	V4 (non-hole)	6	144	37.5 ± 5.8	−4 to −7.88 −6.4 ± 1.1	67%, 20/20 or better	98%, 20/20 or better	79%	97%	0.7%
Sanders et al. (17)	2007	V4 (non-hole)	6	164	37.3 ± 6.0	−3 to −7.88 −6.01 ± 1.40	63%, 20/20 or better	95%, 20/20 or better	85%	97%	0%
Kamiya et al. (18)	2017	V4c (hole)	12	351	34.8 ± 7.4	−0.5 to −5.88 −4.29 ± 1.30	−0.17 ± 0.14 logMAR	−0.21 ± 0.10 logMAR	93%	98%	0%
Pinto et al. (19)	2021	V4c (hole)	12	106	32.4 ± 7.31	−1.88 to −6.00 −4.89 ± 0.99	0.02 ± 0.17 logMAR	−0.01 ± 0.12 logMAR	86%	94%.	0%
Current		V4c, V5 (hole)	12	172	33.3 ± 6.8	−0.38 to −3.0 −2.28 ± 0.56	−0.17 ± 0.12 logMAR	−0.24 ± 0.07 logMAR	91%	100%	0%

*MSE, manifest spherical equivalent; D, diopter; UDVA, uncorrected distance visual acuity; CDVA, corrected distance visual acuity; logMAR, logarithm of the minimal angle of resolution*.

It is clinically essential to compare the visual and refractive outcomes and LASIK complications in a similar degree of myopic correction. Multiple studies have reported results from the initial FDA studies to extensive randomized and meta-analysis studies that show 99.5% of patients achieve 20/40 vision and 90–95% achieve 20/20 vision or better (23). However, LASIK induces a more oblate shape of the cornea than ICL surgery (24, 25). Furthermore, retinal magnification after ICL surgery is known to be reduced to a lesser extent than after LASIK (26, 27). Indeed, we found that ICL implantation induced significantly fewer higher-order aberrations than wavefront-guided LASIK and that contrast sensitivity function was significantly improved after ICL implantation but unchanged after wavefront-guided LASIK for the correction of low-to-moderate myopia. However, the visual and refractive outcomes of ICL surgery are almost comparable with those of LASIK (20). In addition, we also found that hole ICL implantation provided an excellent optical performance, such as modulation transfer function, Strehl ratio, and objective scattering index (28). We also cannot refute the possibility that the cost-effectiveness of LASIK surgery is superior to that of ICL surgery. Moreover, we should be aware that ICL implantation is one of the intraocular surgeries that can be accompanied by endophthalmitis, which is the worst scenario after ICL surgery. The onset rate was estimated to be 0.0167% based on the sizeable online survey of ICL surgeons (29).

Concerning the postoperative complications, we did not observe vision-threatening complications, including symptomatic or asymptomatic cataract formation, pigment dispersion glaucoma, pupillary block, retinal detachment, or significant endothelial cell loss in this series. Moreover, it has been reported that the complications in ICL-implanted eyes for low-to-moderate myopia were almost equivalent to those for high myopia (18, 19). Using the data on a total of 617 eyes with a weighted average follow-up of 13 months, Packer et al. confirmed a 0.49% incidence of asymptomatic anterior subcapsular cataract formation after EVO-ICL implantation (30). Therefore, we assume that the possible risk of current ICL complications in patients with low myopia is essentially equivalent to that in moderate-to-severe myopic eyes.

This study has several limitations. Firstly, the analysis was conducted in a retrospective fashion. Although this is a multicenter study in a successive cohort of patients with low myopia undergoing ICL implantation, a prospective randomized controlled study is ideal to confirm our findings. Secondly, there were some variations in the use of surgical devices and drugs because several experienced surgeons contributed to this multicenter study. We assume that a multicenter study may more accurately reflect the actual status of current ICL surgery than a single-center study because the former may be less affected by their individual surgical skills and experiences than the latter. Thirdly, we enrolled only consecutive patients who completed a 1-year follow-up in this study. As satisfied patients undergoing refractive surgery tended to be lost during routine follow-up, our longitudinal data may have a possible source of selection bias. Fourthly, we included both eyes of the same patient undergoing ICL implantation, but only one eye per patient should be used for statistical analysis. As listed in Supplementary Data 1, we confirmed similar outcomes when only one eye was chosen randomly from each patient. Most published studies on refractive surgery have included both eyes, so we evaluated both eyes when applicable, considering that the number of patients undergoing ICL surgery for low myopia is rather limited. Fifthly, we did not assess the cost-effectiveness of ICL implantation and LASIK in this study. Therefore, it remains unclear and needs to be further elucidated.

## Conclusions

In summary, our multicenter case series confirmed that the ICL performed well to correct low-grade myopia without significant complications during the 1-year observation period. Hence, our findings may support the view that current ICL implantation is one of the viable surgical options for correcting low myopia. This surgical indication may be expanded to include patients with low myopia in the future. However, we should be aware that the long-term outcomes of this new surgical approach currently remain unanswered. More prolonged careful follow-up in a large cohort of patients with low-grade myopia is still necessary to clarify this point.

## Data Availability Statement

The original contributions presented in the study are included in the article/Supplementary Material, further inquiries can be directed to the corresponding author/s.

## Ethics Statement

The study was approved by the Institutional Review Board at Kitasato University Hospital (B21-211) and followed the Tenets of the Declaration of Helsinki. Written informed consent for participation was not required for this study in accordance with the national legislation and the institutional requirements.

## Author Contributions

KK, KS, and AI were involved in the design and conduct of the study. KK, KS, AI, YK, TK, TN, KI, SF, and KF were involved in the collection, management, analysis, and interpretation of data and in the preparation, review, and final approval of the manuscript. All authors contributed to the article and approved the submitted version.

## Conflict of Interest

KS and YK are paid consultants for STAAR Surgical. The remaining authors declare that the research was conducted in the absence of any commercial or financial relationships that could be construed as a potential conflict of interest.

## Publisher's Note

All claims expressed in this article are solely those of the authors and do not necessarily represent those of their affiliated organizations, or those of the publisher, the editors and the reviewers. Any product that may be evaluated in this article, or claim that may be made by its manufacturer, is not guaranteed or endorsed by the publisher.
